# Kinesin-3 motor protein KIF13B localizes to centrosomes and primary cilia and regulates ciliary length and signaling

**DOI:** 10.1186/2046-2530-4-S1-O3

**Published:** 2015-07-13

**Authors:** K B Schou, JB Mogensen, A Aleliunaite, S Morthorst, I Veland, S Christensen, LB Pedersen

**Affiliations:** 1 University of Copenhagen, Copenhagen, Denmark; 2 Cancer Research UK Beatson Institute, Glasgow, UK

## 

*Caenorhabditis elegans *kinesin-3 KLP-6 localises to cilia and regulates ciliary localization of polycystins [1]. Here we investigated if kinesin-3 motors also have cilia-related functions in vertebrates. We used transcriptomics to identify kinesins that are upregulated during serum deprivation and ciliogenesis in mouse fibroblasts and identified *Kif13b *among several kinesin-3 genes that are upregulated concomitantly with ciliogenesis. Western blot and immunofluorescence microscopy confirmed that KIF13B is upregulated during serum deprivation in cultured mammalian cells, and showed that KIF13B localizes to centrosomes and cilia. Analysis of full-length and truncated GFP-KIF13B fusion proteins expressed in retinal pigment epithelial (RPE) cells showed that KIF13B contains at least two centrosome-targeting regions in its central and C-terminal region, respectively. Within the central region of KIF13B we identified by bioinformatics analysis two conserved beta-sandwich fold domains that were identified in mammalian kinesin-3 members KIF13A, KIF1A, KIF1B and *C. elegans *KLP-4 and KLP-6, as well. Immunoprecipitation of ectopically expressed proteins and mass spectrometry analysis indicated that KIF13B interacts with a number of centrosomal proteins, likely via these beta-sandwich fold domains. Depletion of KIF13B in RPE cells using siRNA caused significant elongation of primary cilia, altered IGF1 signaling and decreased expression of Wnt5a.

Our results identify KIF13B as a cilia-associated kinesin involved in regulating ciliary length and signaling, and indicate that KIF13B interacts physically with the centrosome/transition zone. We hypothesize that KIF13B is recruited to the centrosome to regulate its kinesin activity and downstream signaling events and are currently investigating this further.
